# Synthesis of Platinum Complexes from N-Benzyl-2-Aminoethanethiol Derivatives

**DOI:** 10.1100/tsw.2003.31

**Published:** 2003-05-05

**Authors:** Mauro Vieira de Almeida, Laura Lúcia C. de Oliviera, Eloi Teixeira Cesar, Marcus Vinícius N. de Souza

**Affiliations:** ^1^ Departamento de Química, UFJF, 36036-330, Juiz de Fora-MG, Brazil; ^2^ Colégio de Aplicação João XXIII, UFJF, 36036-330, Juiz de Fora-MG, Brazil

**Keywords:** platinum(II) complexes, N-benzyl-2-aminoethanethiol, anticancer agents, cisplatin

## Abstract

Twelve new platinum(II) complexes, analogs of cisplatin, containing a 2-aminoethanethiol N-substituted by several benzyl groups have been prepared and characterized in good yields. The ligands were obtained by reaction between 2-aminoethanethiol hydrochloride and different benzyl halides

## INTRODUCTION

Cisplatin (Fig. 1) is an important drug in the fight against cancer. In association with other types of drugs, it is highly effective in the treatment of different kinds of neoplasias[1,2,3]. The biological activity of cisplatin was discovered by Rosenberg et al. at the end of the 1960s[4]. Due to its side effects, the appearance of resistance, and low solubility[5,6,7], new platinum complexes have been synthesized such as carboplatin, oxaliplatin, and nedaplatin (Fig. 1). Carboplatin shows the same level of activity as cisplatin in the treatment of some kinds of cancer and is much less nephrotoxic and emetic[8]. Oxaliplatin has been approved for the treatment of colorectal cancer in France, and nedaplatin has received approval for use in Japan. Oxaliplatin and nedaplatin have yet to demonstrate significant advantages over cisplatin or carboplatin[9]. This fact shows how it is important to develop new platinum complexes that could effectively act in a larger number of tumors. At the same time, those complexes should present less severe side effects and they should be active in resistant cells lines.

Since some substituted ethylenediamine platinum complexes have shown antitumor activity against a variety of cell tumors[10,11], and also aromatic compounds have shown the possibility of intercalation between DNA bases[12], we synthesized complexes containing a platinum center bound to ethylenediamine derivatives that demonstrated cytotoxicity *in vitro* in carcinoma buccal human cells[13]. Based on those results, we decided to synthesize platinum(II) complexes containing 2-aminoethanethiol N-substituted with several benzyl groups. This choice was made because of the close structural similarity among 2-aminoethanethiol, ethylenediamine, and cysteine.

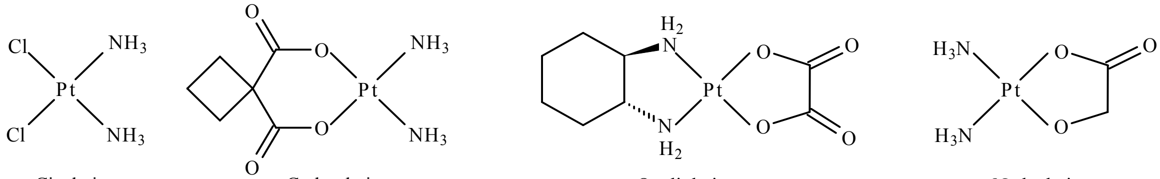

Cisplatin Carboplatin Oxaliplatin Nedaplatin

**Figure 1**

## RESULTS AND DISCUSSION

The ligands **6–10** were prepared in satisfactory yields (50–100%) by the reaction of 2-aminoethanethiol hydrochloride and sodium bicarbonate with the corresponding benzyl halide **1-5** in ethanol, in a range varying between 70 h to 7 days (Scheme 1). $^{1}H$ NMR of the ligands showed signals between $\delta$ 2.6–2.7, 3.0–3.1, 3.6–3.9, and 7.1–8.4 corresponding to the $C\underline{H}_2SH$, $C\underline{H}_2NH$, benzyl $C\underline{H}_2$ and the aromatic hydrogens, respectively. In the $^{1}H$ NMR spectrum of compound **9,** a signal at $\delta$ 3.67, attributable to the $OC\underline{H}_3$, was also noticed. $^{13}C$ NMR spectra showed signals between $\delta$ 28.0–35.0, 34.4–35.4, 38.5–39.4, and 114.1–159.2, corresponding to the methylenic carbons $\underline{C}H_2SH$, $\underline{C}H_2NH$, benzyl $\underline{C}H_2$, and aromatic carbons. Furthermore, a signal at $\delta$ 55.1 attributable to the $O\underline{C}H_3$ of compound **9** was also found. The IR spectra of these ligands showed absorptions corresponding to aromatic CH, NH, and SH at 3033–2907, 2950–3065, and 2550–2588 $cm^{-1}$, respectively. For compounds **8** and **10**, absorptions attributable to $NO_2$ at 1509, 1245 $cm^{-1}$ and 1450, 1347 $cm^{-1}$ were also noticed.

The dichloro platinum(II) complexes **11–15** (65–88% yield) were synthesized by the reaction of these ligands with $K_2[PtCl_4]$ in water at room temperature for 24 h and isolated by filtration. For the complexes, one can see in their IR spectra absorptions corresponding to $\gamma$ Pt-N, $\gamma$ Pt-S, and $\gamma$ Pt-Cl between 500–550, 440–476, and 300–338 $cm^{-1}$, respectively, in addition to the absorptions observed for the ligands. In the $^{1}H$ NMR spectrum of these complexes, signals were observed between $\delta$ 2.1–2.8 and 3.0–3.1, corresponding to the $C\underline{H}_2SH$ and $C\underline{H}_2NH$, signals between $\delta$ 4.0–4.8 in the case of benzyl $C\underline{H}_2$, and finally signals in the region of $\delta$ 7.1–8.2 for the aromatic hydrogens. The compound **14** also showed a signal at $\delta$ 3.8 attributable to the $OCH_3$.

The compounds **6–10** were reacted with an equimolar amount of *in situ* generated potassium tetraiodoplatinate(II) to produce diiodo platinum(II) complexes **16–20**, in 89–100% yields. Besides the absorptions observed in the spectra of the ligands, the IR spectra of the iodine complexes **16–20** showed $\gamma$ Pt-N and $\gamma$ Pt-S between 527–547 and 435–468 $cm^{-1}$, respectively. $^{1}H$ NMR spectra of these complexes showed signals between $\delta$ 2.4–2.7, 2.7–3.3, 4.3–4.8, and 7.0–8.8 corresponding to the $C\underline{H}_2SH$, $C\underline{H}_2NH$, benzyl $C\underline{H}_2$, and the aromatic hydrogens. In the case of $^{13}C$ NMR, signals between $\delta$ 36.0–58.0 and 123.0–139.0 corresponding to the methylenic carbons $\underline{C}H_2SH$, $\underline{C}H_2NH$, benzyl $\underline{C}H_2$, and aromatic carbons were observed.

The complexes **21** and **22** were prepared in order to compare their cytotoxic activity in relation to the complexes **11–20**, containing N-benzyl groups. They were synthesized by the same procedure described above for the complexes **11–20** in 84 and 82% yield. IR spectra of the complex **21** and **22** showed absorptions corresponding to NH and CH aliphatic at 3193 and 2929 $cm^{-1}$, respectively. $^{1}H$ NMR signals were observed at $\delta$ 2.8 corresponding to $C\underline{H}_2SH$ and $C\underline{H}_2NH$, respectively.

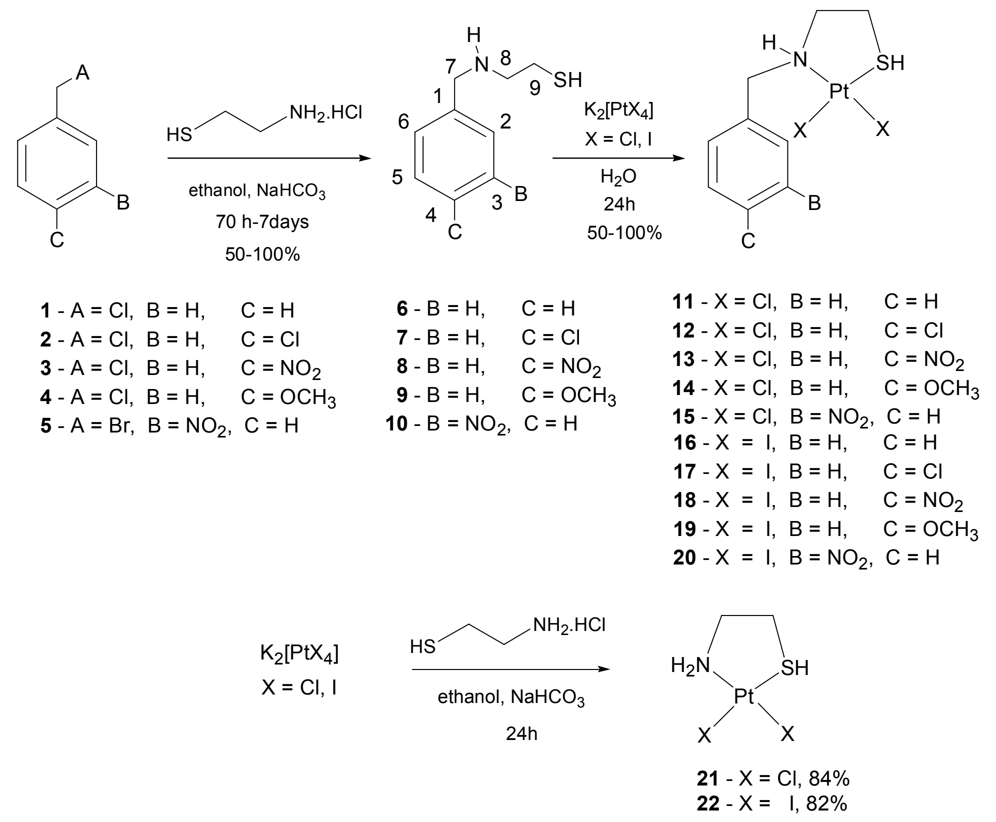

**Scheme 1**

## MATERIAL AND EQUIPMENT

IR spectra were obtained on a Bomem FT IR MB-102 spectrometer in CsI pellets. $^{1}H$ NMR (200 and 400 MHz) and $^{13}C$ NMR (50 and 100 MHz) spectra were recorded on Brucker Avance DRX 200 and DRX 400 spectrometers at the Federal University of Minas Gerais. Column chromatography was performed using silica gel 60G (0.063–0.200 mm and 0.2–0.5 mm, E. Merck). Elemental analyses were carried at Laboratoire Central de Microanalyse du ICSN-CNRS, Gif-sur-Yvette, France.

*Reagents:* All chemicals were analytical grade and used without further purification.

## METHOD

### Synthesis of Ligands 6, 7, 8, 9, and 10: General Procedure

To a solution of 2-aminoethanethiol hydrochloride (12 mmols) in ethanol (10 mL) and sodium bicarbonate (12 mmols), the corresponding benzyl halide **1**, **2**, **3**, **4,** or **5** (10 mmols) were added during 4 h. The reaction mixture was stirred at room temperature for 72 h (to ligands **6** and **9**), 70 h (to ligands **7** and **10**), and 7 days (to ligand **8**). Then, it was evaporated under reduced pressure and the residue purified on silica gel 60 G (0.2–0.5 mm). The ligands **6**, **7,** and **10** were purified by recrystallization from ethanol/ether. Yields: 1.62 g, 97% for the ligand **6**; 1.96 g, 97% for the ligand **7**; 2.14 g, 79% for the ligand **8**; 1.18 g, 50% for the ligand **9**; 2.71g, 100% for the ligand **10**.

**6**: as a white crystal mp 131°C (from ethanol/ether); IR νmax CsI ($cm^{-1}$): 2989, 2957, 2907, 2584, 1600, 1477, 1404, 1244, 1110, 899, 768, 703; $^1H$ NMR (200 MHz, $D_2O$) δ: 2.65 (t, 2H, H9, $J_{9,8}$ = 6.7 Hz); 3.01 (t, 2H, H8, $J_{8,9}$ = 6.7 Hz); 3.72 (s, 2H, H7); 7.31 (m, 5H, Ph); $^{13}C$ NMR (50.33 MHz, $D_2O$) δ: 28.1 (C9); 35.1 (C8); 38.5 (C7); 127.9 (C4); 129.3 (C2, C3, C5, C6); 138.4 (C1).

**7**: as a white crystal mp 133°C (from ethanol/ether); IR νmax CsI ($cm^{-1}$): 2992, 2916, 2582, 1592, 1490, 1404, 1450, 1091, 893, 839; $^1H$ NMR (200 MHz, $D_2O$) δ: 2.66 (t, 2H, H9, $J_{9,8}$ = 5.7 Hz); 3.04 (t, 2H, H8, $J_{8,9}$ =5.7 Hz); 3.68 (s, 2H, H7); 7.32 (m, 4H, Ph); $^{13}C$ NMR (50.33 MHz, $D_2O$) δ: 28.0 (C9); 34.4 (C8); 38.5 (C7); 129.1 (C2, C6); 130.8 (C3, C5); 132.9 (C1); 139.4 (C4).

**8**: as an oil; Anal. Calcd. for $C_9H_{12}N_2O_2S.2HCl$: C, 37.90; H, 4.95; N, 9.82; Found: C, 38.20; H, 5.17; N, 9.53; IR νmax CsI ($cm^{-1}$): 2897, 2683, 2582, 1600, 1510, 1351, 1245, 1109, 853, 799, 709; $^1H$ NMR (200 MHz, $C_5D_5N$) δ: 3.05 (t, 2H, H9, $J_{8,9}$ = 6.7 Hz); 3.57 (t, 2H, H8, $J_{8,9}$ = 6.7 Hz); 3.96 (s, 2H, H7); 7.60 (d, 2H, H6, H2, J= 8.5 Hz); 8.15 (d, 2H, H3, H5, J= 8.5 Hz); $^{13}C$ NMR (50.33 MHz, $C_5D_5N$) δ: 29.2 (C9); 34.9 (C8); 39.2 (C7); 129.8; 130.3; 135.0; 147.5 (C1-C6).

**9**: as an oil; IR νmax CsI ($cm^{-1}$): 3436, 2959, 1609, 1508, 1468, 1239; $^1H$ NMR (200 MHz, $C_5D_5N$) δ: 2.72 (t, 2H, H9, $J_{8,9}$ = 6.7 Hz); 3.08 (t, 2H, H8, $J_{8,9}$ = 6.7 Hz); 3.67 (s, 3H, O$C\underline{H}_3$); 3.77 (s, 2H, H7); 7.34 (d, 2H, H6, H2, J = 8.5 Hz); 6.95 (d, 2H, H3, H5, J = 8.5 Hz); $^{13}C$ NMR (50.33 MHz, $C_5D_5N$) δ: 35.0 (C9); 35.4 (C8); 39.3 (C7); 55.1 (O$\underline{C}H_3$); 114.2 (C2, C6); 130.5 (C3, C5); 131.1 (C1); 159.1 ($\underline{C}OCH_3$).

**10**: as a white crystal mp 75°C (from ethanol/ether); Anal. Calcd. for $C_9H_2N_2O_2S.2HCl.H_2O$: C, 35.65; H, 5.32; N, 9.24; Found: C, 35.45; H, 5.07; N, 9.13; IR νmax CsI ($cm^{-1}$): 3065, 3033, 2924, 2872, 2573, 1531, 1450, 1348, 1127, 930, 811, 709; $^1H$ NMR (200 MHz, $D_2O$) δ: 2.77 (t, 2H, H9, $J_{9,8}$= 6.5 Hz); 3.01 (t, 2H, H8, $J_{8,9}$ = 6.5 Hz); 3.89 (s, 2H, H7); 7.65 (t, 1H, H5, $J_{5,6}$ = 7.8 Hz); 7.75 (d, 1H, H6, $J_{6,5}$ = 7.8 Hz); 8.04 (d, 1H, H4, $J_{8,9}$ = 7.8 Hz); 8.12 (s, 1H, H2) ; $^{13}C$ NMR (50.33 MHz, $D_2O$) δ: 28.0 (C9); 34.4 (C8); 38.5 (C7); 112.8 (C2); 124.0 (C1); 130.2 (C3); 136.1 (C5); 140.4 (C6); 148.4 (C4).

## Synthesis of Complexes 11–15 and 21: General Procedure

The appropriate ligand (1 mmol) was dissolved in water (5 mL) and added slowly with stirring to a solution of $K_2PtCl_4$ (415 mg, 1 mmol) in water (10 mL). After 24 h in the dark at room temperature, the yellow solid formed was filtered off, washed with water, and dried. In the preparation of the complex **21**, the ligand 2-aminoethanothiol hydrochloride and sodium bicarbonate (84 mg, 1 mmol) were previously dissolved in water (5 mL). Yields: 76% for **11**, 88% for **12**, 76% for **13**, 66% for **14**, 80% for **15**, 84% for **21.**

**11**: as a yellow solid; Anal. Calcd. for $C_9H_{13}NCl_2SPt.H_2O$: C, 24.95; H, 3.03; N, 3.23; Found: C, 24.77; H, 2.98; N, 3.21; IR νmax CsI ($cm^{-1}$): 3174, 3085, 2977, 1494, 1455, 1242, 1161, 1073, 1002, 768, 534, 476, 461, 316; $^1H$ NMR (200 MHz, DMSO-*d₆*) δ: 2.72 (m, 2H, H9); 3.20 (m, 2H, H8); 4.40 (m, 2H, H7); 7.70 (m, 5H, Ph).

**12**: as a yellow solid; Anal. Calcd. for $C_9H_{12}NCl_3SPt.H_2O$: C, 22.25; H, 2.90; N, 2.88; S, 6.60; Found: C, 21.90; H, 2.63; N, 2.63; S, 6.29; IR νmax CsI ($cm^{-1}$): 3081, 2976, 2928, 1491, 1408, 1091, 1016, 842, 763, 654, 534, 506, 463, 318; $^1H$ NMR (200 MHz, DMSO-$d_6$) δ: 2.60 (m, 2H, H9); 2.90 (m, 2H, H8); 4.4 (m, 2H, H7); 7.6 (m, 4H, Ph).

**13**: as a yellow solid; Anal. Calcd. for $C_9H_{12}N_2Cl_2O_2SPt.2H_2O$: C, 21.02; H, 3.14; N, 5.44; Found: C, 20.65; H, 2.97; N, 4.96; IR νmax CsI ($cm^{-1}$): 3182, 3058, 2928, 1603, 1520, 1352, 1245, 1106, 857, 708, 536, 465, 317; $^1H$ NMR (200MHz, DMSO-$d_6$) δ: 2.70 (m, 2H, H9); 3.00 (m, 2H, H8); 4.40 (d, 1H, H7′, $J_{7,NH}$ = 15 Hz); 4.60 (d, 1H, H7, $J_{7',NH}$ = 13.5 Hz); 8.37 (d, 2H, H6, H2, J = 8.4 Hz); 8.20 (d, 2H, 2H, H3, H5, J = 8.4 Hz).

**14**: as a yellow solid; Anal. Calcd. for $C_{10}H_{15}NCl_2OSPt.2H_2O$: C, 24.05; H, 3.84; N, 2.80; S, 6.80; Found: C, 23.96; H, 3.85; N, 2.77; S, 6.80; IR νmax CsI ($cm^{-1}$): 3176, 3088, 2973, 2834, 1608, 1513, 1248, 1178, 1030, 840, 548, 523, 469, 317; $^1$H NMR (200 MHz, DMSO-$d_6$) δ: 2.60 (m, 2H, H9); 3.00 (m, 2H, H8); 4.40 (m, 2H, H7); 7.40 (d, 2H, H2, H6, J= 8.5 Hz); 7.56 (d, 2H, H3, H5, J= 8.5 Hz).

**15**: as a yellow solid; Anal. Calcd. for $C_9H_{12}N_2Cl_2O_2SPt.H_2O$: C, 21.78; H, 2.43; N, 5.46; S, 6.46; Found: C, 21.61; H, 2.62; N, 5.43; S, 6.08; IR νmax CsI ($cm^{-1}$): 3210, 3181, 3095, 2973, 2934, 1537, 1352, 1143, 1000, 805, 714, 669, 534, 489, 472, 314; $^1$H NMR (200 MHz, DMSO-$d_6$) δ: 2.30 (m, 2H, H9); 2.70 (m, 2H, H8); 4.20 (m, 2H, H7); 8.6 (m, 4H, Ph).

**21**: as a yellow solid; Anal. Calcd. for $C_2H_7Cl_2NSPt$: C, 7.00; H, 2.06; N, 4.08; S, 9.34; Found: C, 6.74; H, 2.27; N, 3.97; S, 8.89; IR νmax CsI ($cm^{-1}$): 3196, 3110, 2929, 1577, 1238, 984.

## Synthesis of Complexes 16–20 and 22: General Procedure

A solution of $K_2PtCl_4$ (415 mg, 1 mmol) and KI (664 mg, 4 mmol) in water (10 mL) was stirred in the dark at room temperature for 30 min, after which the appropriate ligand (1 mmol) dissolved in water (5 mL) was added slowly. After stirring in the dark at room temperature for 24 h, the brown product was isolated by filtration and recrystalized from acetone/water. In the preparation of the complex **22** the ligand 2-aminoethanothiol hydrochloride and sodium bicarbonate (1 mmol) were previously dissolved in water (5 mL). Yields: 96% for **16**, 100% for **17**, 89% for **18**, 91% for **19**, 98% for **20**, 82% for **22**.

**16**: as a brown solid mp 212°C (from water/acetone); Anal. Calcd. for $C_9H_{13}NI_2SPt$: C, 17.54; H, 2.13; N, 2.27; Found: C, 17.81; H, 2.22; N, 2.12; IR νmax CsI ($cm^{-1}$): 3230, 3175, 3094, 3024, 1494, 1453, 1414, 1231, 1137, 986, 770, 700, 532, 643, 464; $^1$H NMR (400 MHz, $C_3D_6O$) δ: 2.70 (m, 4H, H8, H9); 4.24 (d, 1H, H7, $J_{7,NH}$ = 10 Hz ); 4.39 (d, 1H, H7′, $J_{7',NH}$ = 10 Hz); 7.30 (m, 3H, Ph); 7.45 (d, 2H, H2, H6, J= 3.7 Hz); 7.47 (m, 3H, H3, H5); $^{13}$C NMR (100 MHz, $C_3D_6O$) δ: 39.1 (C9); 41.2 (C8); 50.2 (C7); 128.8; 129.4; 130.4; 134.7 (Ph).

**17**: as a brown solid mp 233°C (from water/acetone); Anal. Calcd. for $C_9H_{12}NclI_2SPt$: C, 16.61; H, 1.86; N, 2.15; Found: C, 16.41; H, 1.95; N, 2.03; IR νmax CsI ($cm^{-1}$): 3168, 3095, 3025, 2962, 2922, 1572, 1491, 1228, 1136, 1087, 1014, 836, 725, 621, 540, 504, 465; $^1$H NMR (400 MHz, $C_3D_6O$) δ: 2.60 (m, 2H, H9); 2.80 (m, 2H, H8); 4.40 (d, 2H, H7, H7′, $J_{7,7'}$= 11.7 Hz); 7.67 (d, 2H, H2, H6, J= 8.5 Hz); 7.57 (d, 2H, H3, H5, J= 8.5 Hz); $^{13}$C NMR (50.33 MHz, $C_3D_6O$) δ: 39.7 (C9); 40.7 (C8); 50.5 (C7); 129.6; 129.9; 132.7 (Ph).

**18**: as a brown solid mp 234°C (from water/acetone); Anal. Calcd. for $C_9H_{12}N_2O_2I_2SPt$: C, 15.35; H, 1.83; N, 4.23; Found: C, 15.41; H, 1.24; N, 3.57; IR νmax CsI ($cm^{-1}$): 3234, 3187, 3111, 3059, 2973, 1592, 1517, 1410, 1347, 1126, 845, 729, 524, 472; $^1$H RMN (400 MHz, $C_3D_6O$) δ: 2.80 (m, 4H, H8, H9); 4.57 (d, 1H, H7′, $J_{7,NH}$= 13.6 Hz ); 4.64 (d, 1H, H7, $J_{7',NH}$= 13.6 Hz); 7.95 (d, 2H, H5, H6, J= 4.5 Hz); 8.30 (d, 2H, H2, H3, J= 4.5 Hz); $^{13}$C NMR (50.33 MHz, $C_3D_6O$) δ: 40.2 (C9); 40.4 (C8); 51.0 (C7); 124.8; 130.0; 132.3 (Ph).

**19**: as a brown solid mp 218°C (from water/acetone); Anal. Calcd. for $C_{10}H_{15}NOI_2SPt.0.5H_2O$: C, 18.59; H, 2.34; N, 2.17; Found: C, 18.32; H, 2.29; N, 2.14; IR νmax CsI ($cm^{-1}$): 3175, 3085, 2964, 2834, 1608, 1511, 1247, 1178, 1028, 1014, 842, 748, 522, 453.

**20**: as a brown solid mp 238°C (from water/acetone); Anal. Calcd. for $C_9H_{12}N_2O_2I_2SPt$: C, 16.35; H, 1.83; N, 4.23; Found: C, 16.49; H, 1.96; N, 3.89; IR νmax CsI ($cm^{-1}$): 3290, 3235, 3059, 2930, 1566, 1410, 1349, 1138, 904, 805, 679, 541, 462; $^1$H NMR (400 MHz, $C_3D_6O$) δ: 3.00 (m, 4H, H8, H9); 4.62 (d, 1H, H7′, $J_{7,NH}$ = 10 Hz); 4.70 (d, 1H, H7, $J_{7',NH}$= 13 Hz); 7.76 (t, 1H, H5, $J_{6,4}$= 8 Hz); 8.15 (d, 1H, H6, $J_{6,5}$ = 8 Hz); 8.28 (d, 1H, H4, $J_{4,5}$ = 8 Hz ); 8.50 (s, 1H, H2);

$^{13}$C NMR (100 MHz, $C_3D_6O$) δ: 40.0 (C8, C9); 50.0 (C7); 124.1; 125.6; 126.7; 134.7 (Ph); 131.2 (C1); 137.3 (C4).

**22**: as a brown solid mp 282°C (from water/acetone); Anal. Calcd. for $C_2H_7NOI_2SPt$: C, 5.57; H, 1.34; N, 2.66; S, 6.09 Found: C, 5.79; H, 1.29; N, 2.95; S, 5.72; IR νmax CsI ($cm^{-1}$): 3185, 3099, 3025, 2922, 1656, 1442, 1224, 978, 846; $^{1}$H NMR (400 MHz, $C_3D_6O$) δ: 2.80 (m, 4H, C$\underline{H}_2$S, C$\underline{H}_2$N).

## ACKNOWLEDGMENTS

We wish to thank Drs. R. Grazul, A.P.S. Fontes, and M. Le Hyaric for many helpful discussions. E.T.C. and L.L.C.O. gratefully acknowledge CAPES (Brazil) for a fellowship.
